# Evaluation of oral immunotherapy efficacy and safety by maintenance dose dependency: A multicenter randomized study

**DOI:** 10.1016/j.waojou.2020.100463

**Published:** 2020-09-29

**Authors:** Kiyotake Ogura, Noriyuki Yanagida, Sakura Sato, Takanori Imai, Komei Ito, Naoyuki Kando, Masanori Ikeda, Rumiko Shibata, Yoko Murakami, Takao Fujisawa, Mizuho Nagao, Norio Kawamoto, Naomi Kondo, Atsuo Urisu, Ikuya Tsuge, Yasuto Kondo, Kazuko Sugai, Osamu Uchida, Mitsuyoshi Urashima, Masami Taniguchi, Motohiro Ebisawa

**Affiliations:** a Department of Pediatrics, National Hospital Organization, Sagamihara National Hospital, Kanagawa, Japan; b Course of Allergy and Clinical Immunology, Juntendo University Graduate School of Medicine, Tokyo, Japan; c Department of Allergy, Clinical Research Center for Allergy and Rheumatology, National Hospital Organization, Sagamihara National Hospital, Kanagawa, Japan; d Department of Pediatrics, Showa University School of Medicine, Tokyo, Japan; e Department of Allergy, Aichi Children's Health and Medical Center, Aichi, Japan; f Department of Pediatrics, National Hospital Organization, Fukuyama Medical Center, Hiroshima, Japan; g Department of Pediatric Acute Medicine, Okayama University Graduate School of Medicine, Dentistry and Pharmaceutical Sciences, Okayama, Japan; h Department of Pediatrics, National Hospital Organization, Fukuoka National Hospital, Fukuoka, Japan; i Institute for Clinical Research, National Hospital Organization, Mie National Hospital, Mie, Japan; j Department of Pediatrics, Graduate School of Medicine, Gifu University, Gifu, Japan; k Department of Pediatrics, Fujita Health University, The Second Teaching Hospital, Aichi, Japan; l Department of Pediatrics, National Hospital Organization, Yokohama Medical Center, Kanagawa, Japan; m Division of Molecular Epidemiology, The Jikei University School of Medicine, Tokyo, Japan

**Keywords:** Food hypersensitivity, Immunotherapy, Dose-response relationship, Desensitization, FA, food allergy, HE, hens’ egg, CM, cow’s milk, OIT, oral immunotherapy, OFC, oral food challenge, sIgE, specific immunoglobulin E, sIgG, specific immunoglobulin G, sIgG_4_, specific immunoglobulin G_4_, SLIT, sublingual immunotherapy, EPIT, epicutaneous immunotherapy, SU, sustained unresponsiveness, StU, short-term unresponsiveness

## Abstract

**Background:**

Generally, oral immunotherapy (OIT) aims for daily administration. Recently, the efficacy of treatment with OIT at a low dose has been reported. However, the optimal dose and the evaluation of dose-dependent OIT outcome have not been described.

**Methods:**

A multicenter, parallel, open-labeled, prospective, non-placebo controlled, randomized study enrolled 101 Japanese patients for treatment with OIT. We hypothesized that target dose OIT would induce short-term unresponsiveness (StU) earlier than reduced dose OIT. StU was defined as no response to 6200 mg whole egg, 3400 mg milk, and 2600 mg wheat protein, as evaluated by oral food challenge after 2-week ingestion cessation. To compare the two doses of OIT efficacy, the maximum ingestion doses during the maintenance phase of OIT were divided into 100%-dose or 25%-dose groups against their target StU dose, respectively. A total of 51 patients were assigned to the 100%-dose group [hen's egg (HE) = 26, cow's milk (CM) = 13, wheat = 12] and 50 to the 25%-dose group (HE = 25, CM = 13, wheat = 12). Primary outcome was established by comparing StU at 1 year. Secondary outcome was StU at 2 years and established by comparing allergic symptoms and immunological changes.

**Results:**

The year 1 StU rates (%) for the 100%- and 25%-dose groups were 26.9 vs. 20.0 (HE), 7.7 vs. 15.4 (CM), and 50.0 vs. 16.7 (wheat), respectively. The year 2 StU rates were 30.8 vs. 36.0 (HE), 7.7 vs. 23.1 (CM), and 58.3 vs. 58.3 (wheat), respectively. There were no statistically significant differences in StU between years 1 and 2. The total allergic symptom rate in the 25%-dose group was lower than that in the 100%-dose group for egg, milk, and wheat. Antigen-specific IgE levels for egg-white, milk, and wheat decreased at 12 months.

**Conclusions:**

Reduced maintenance dose of egg OIT showed similar therapeutic efficacy to the target dose. However, we were not able to clearly demonstrate the efficacy, particularly for milk and wheat. Reducing the maintenance dose for eggs, milk, and wheat may effectively lower the symptoms associated with their consumption compared to the target OIT dose. Furthermore, aggressive reduction of the maintenance dose might be important for milk and wheat, compared to the 25%-dose OIT.

**Trial registration:**

UMIN000009373, Multicenter Oral Immunotherapy for Hen's Egg, Cow's Milk, and Wheat-Allergic Children at Outpatient Clinic.

## Introduction

Food allergy (FA) is a major health issue that has been increasing at the global level.^1^ Based on a national survey, Imai et al reported that hen's egg (HE; 39.0%), cow's milk (CM; 21.8%), and wheat (11.7%) represent the most frequent causative food allergens in Japan.^2^ Accordingly, numerous reports are available on oral immunotherapy (OIT) for HE,^3^, ^4^, ^5^ CM,^3^^,^^6^, ^7^, ^8^, ^9^ wheat,^10^, ^11^, ^12^, ^13^ and peanut^14^, ^15^, ^16^, ^17^, ^18^, ^19^, ^20^ allergies. Meanwhile, other studies^21^, ^22^, ^23^ have reported that patients treated with OIT reached a state of desensitization or sustained unresponsiveness (SU) providing an overall improvement in their quality of life. These breakthrough findings suggest that the efficacy of OIT may be considered a beacon of hope for patients with severe FA.

Initially, OIT aims for a daily tolerant dose that is increased rapidly; however, it is difficult to prevent adverse allergic reactions during OIT.^24^ Thus, several studies have attempted to address these issues using modified OIT. For example, omalizumab administration combined with OIT^25^ was used to decrease adverse reactions, and sublingual immunotherapy (SLIT) combined with OIT was used to safely increase symptom threshold.^26^ Additionally, epicutaneous immunotherapy (EPIT) offers another novel safe approach for increasing the symptom threshold.^27^^,^^28^ Recently, OIT initiated at a low dose has been used to improve the safety of allergen ingestion for patients with severe egg, milk, and peanut allergies.^22^^,^^29^, ^30^, ^31^ However, particularly in children, OIT may cause stress over long-term treatment in proportion to the amount of ingested allergen.

An alternative strategy to enhance OIT safety is to attempt to achieve tolerance for FA through administration of food allergens at low doses. This is considered a worthwhile pursuit, considering that little evidence^32^, ^33^, ^34^ is available regarding the ideal maintenance dose for causative allergens during OIT. In 2012, a comparison of dose-dependent OIT for CM allergy was reported by Keet et al.^32^ The authors found that 2 different maintenance doses of CM OIT (1000 and 2000 mg) exhibit similar effectiveness, although this efficacy was evaluated in combination with SLIT. Furthermore, in 2017, an evaluation of dose-dependent OIT for peanut allergy was reported by Vickery et al,.^33^ demonstrating that low dose peanut OIT (300 mg/day) achieved similar SU to those achieved by high dose (3000 mg/day) treatment. Similarly, in 2019, a cross-over OIT for wheat allergy was reported by Nowak-Węgrzyn et al.^34^ The patients in the two OIT dose groups (1445 mg/day and 2748 mg/day) achieved desensitization in a similar manner. However, previous dose-dependent studies described narrow dose-differences for CM and wheat allergens during OIT. In addition, to the best of our knowledge, no study has characterized the optimal ingestion dose for HE allergens during OIT. Thus, it remains uncertain whether SU can be obtained with a lower OIT dose, or whether target dose OIT strongly induces SU. To clarify this issue, this study compared 2 maintenance OIT doses, target and lower doses, capable of inducing short-term unresponsiveness (StU). Although traditional OIT studies measured long SU up to 4–10 weeks after cessation of therapy,^4^^,^^16^^,^^25^^,^^26^^,^^32^, ^33^, ^34^ we defined the alternative outcome as StU after 2-week cessation of OIT. This new approach to OIT can be applied to various cases of food allergens frequently present in patients’ diets. Moreover, this study represents the first description of applying four times dose-differences of OIT outcomes for HE, CM, and wheat capable of inducing unresponsiveness earlier in patients with moderate allergies; meanwhile the low dose applied herein was lower than that reported previously.^32^^,^^34^

## Methods

### Study design

This was a randomized, prospective, non-placebo controlled, multicenter, open-label, and parallel-group study conducted in Japan. This study included 9 allergy units in separate hospitals and was supported by a statistician. The enrollment period was from December 2012 to April 2014. The follow-up period extended to April 2016.

### Inclusion criteria

Patients were assessed for eligibility based on their positive objective and significantly subjective symptoms for HE, CM, or wheat allergies at the baseline oral food challenge (OFC) performed by allergists. The inclusion criteria were: (1) patients of 3–15 years of age; (2) patients with positive OFC test result within 3 months before the trial; and (3) patients allergic to either HE, CM, or wheat.

All patients had supported their allergic sensitization, as shown by serological antigen-specific IgE of > 0.35 (kU_A_/L). A confirmatory skin prick test was not performed. The immediate type of FA was defined as having at least 1 or more objective allergic symptoms such as obvious urticaria, repetitive cough, wheezing, emesis, and diarrhea, or clinically significant subjective symptoms such as throat pain, abdominal pain, and other changes in activity, confirmed by OFC within 3 months before the study, and evaluated by an allergist. The thresholds for eliciting positive symptoms for HE, CM, or wheat-allergic children were > 194 mg of whole egg protein (one thirty-second amount of scrambled egg) and ≤ 1550 mg (one quarter of a scrambled egg); > 102 mg of milk protein (3 mL of unheated milk) and ≤ 850 mg (25 mL of unheated milk); or > 78 mg of wheat protein (1.5 g of boiled pasta or 3 g of boiled udon noodle) and ≤ 650 mg (12.5 g of boiled pasta or 25 g of boiled udon noodle), respectively. These lower and upper limits were set to reduce the risk of anaphylaxis in outpatients while still ensuring statistically clear differences.

### Exclusion criteria

The exclusion criteria were: (1) patients with absence of objective or significantly subjective allergic symptoms (eg, only slight subjective symptoms: temporary discomfort of the oral cavity, throat, and abdomen) at the baseline OFC; (2) patients who experienced anaphylaxis and were treated with adrenaline injection at the baseline OFC just before the trial (for safety reasons and to make the differences in the outcome clear); (3) patients with “not well-controlled” atopic dermatitis, bronchial asthma, or any underlying disease; (4) patients who had been treated with some other immunotherapy (eg, SLIT, another OIT); or (5) patients with a developmental problem or mental disorder (eg, restless patients, to reduce the risk of exercise-induced anaphylaxis just after OIT ingestion at home). However, we did not exclude patients based on the level of antigen-specific IgE, or past history of anaphylaxis.

### Protocol for OIT

OIT target doses were defined as ingestion of 6200 mg of whole egg protein (a scrambled egg, made using 50 g of raw egg), 3400 mg of CM protein (100 mL of unheated milk), or 2600 mg of wheat protein (50 g of boiled pasta or 100 g of boiled udon noodle).^35^ The maximum ingestion doses at the maintenance phase of OIT were divided into the 100%-dose or 25%-dose groups against their target StU dose. This target dose of egg was similar to the full dose of OFC (Table S1), with reference to the Japanese pediatric guideline for FA.^36^ We utilized such a large difference between groups to determine whether different doses of a food allergen induce StU. The target doses of CM and wheat were decreased to nearly half the full dose. However, a potential difficulty was identified for pre-school children to ingest a half dose of raw milk or wheat noodles during OIT. This was postulated to affect the adherence to the OIT, as the patients were requested to consume a large volume with only small amounts of protein in the wheat noodles and raw milk compared to scrambled eggs. For instance, 100 mL of raw milk and 100 g of udon noodles are large of volumes for young children to consume every day. Nevertheless, we did not permit the patients to ingest alternate food options (eg, milk cake) during the build-up phase in order to avoid the inaccurate measurement of the causative foods by the parents. The only exception to this, was for the wheat OIT, in which the patients were allowed to consume alternative pasta or bread foods that contained the same amount of protein as the udon noodles, after reaching the maintenance dose with permission of the attending allergist.

**Fig. 1:**
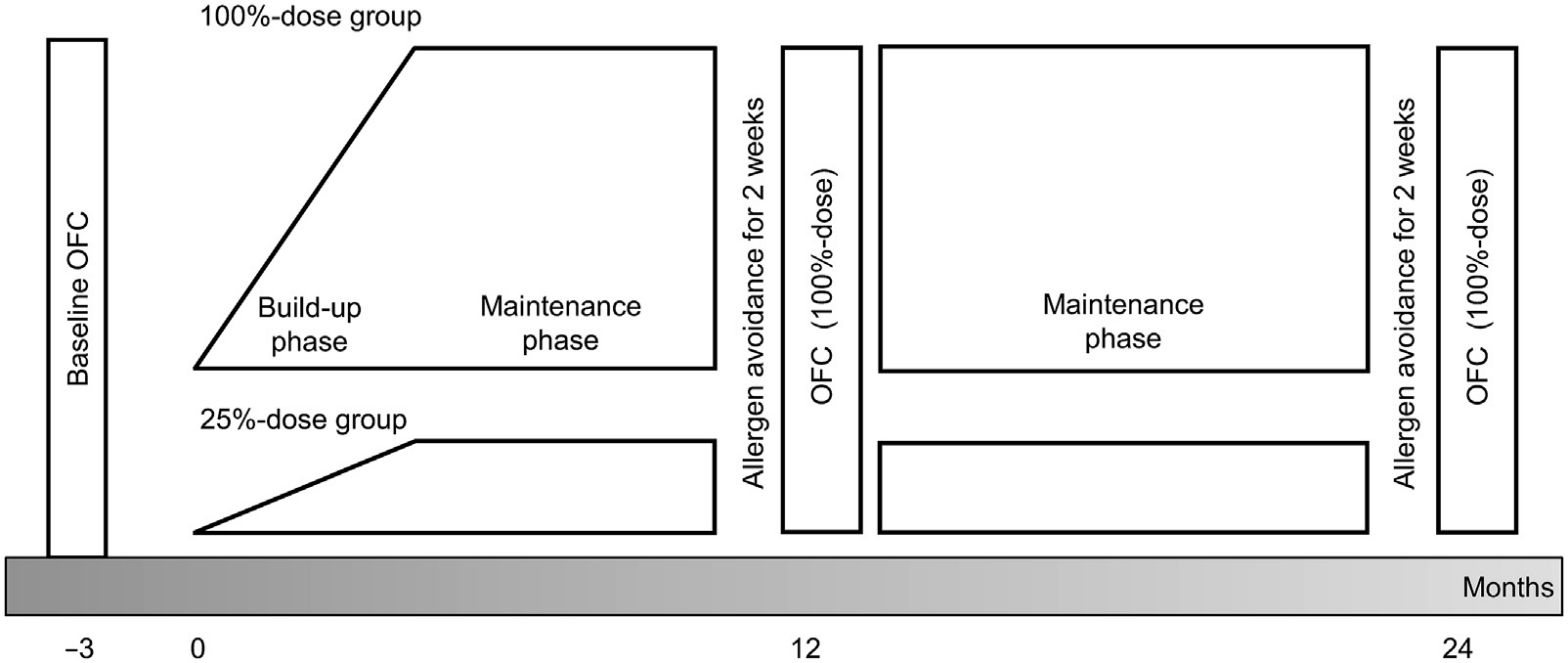
Study protocol. OFC: oral food challenge. Patients consenting to oral immunotherapy were randomized into 2 groups. At-home-ingestion dose gradually increased, if the allergic symptoms did not occur for more than 5 consecutive days. (build-up; Y axis). OIT target doses were defined as ingestion of 6200 mg of whole egg protein, 3400 mg of CM protein, 2600 mg of wheat protein. During the build-up and maintenance phase, the maximum dose ingestion in 100%-OIT group was same as the target dose. The maximum dose in 25%-OIT group was limited to 25% of the target dose. Build-up and maintenance phases occurred at outpatient clinics. After 2 weeks of complete causative food avoidance, OFC for short-term unresponsiveness was performed at 12 and 24 months (X axis)

The patients who consented to OIT were randomly assigned to the 2 groups, initially at a 1:1 ratio (Fig. 1). The stratified factor of randomization was the threshold of objective, and significantly subjective, symptoms at baseline OFC. The patients were asked to consume their respective allergens daily at home. These causative foods for OIT were purchased by the patients themselves at the market. Patients used an accurate scale that could measure 0.1–0.01 g of scrambled egg or boiled udon noodle, or 0.1–0.01 mL of unheated milk with guidance from the medical doctors and dietitians. The ingestion doses of OIT were gradually increased (Table S2) at the outpatient clinic or at home, to a maintenance dose if the allergic symptoms did not occur for more than 5 consecutive days. A total of 20 increasing dose levels were assigned for the egg and 25 for the milk and wheat. For asymptomatic individuals, a minimum of 100 days for the egg, and 125 days for the milk and wheat was required to reach the maintenance dose. The patients were advised to rest for more than 1 h after OIT ingestion to reduce the risk of exercise-induced adverse allergic reactions. The gradual ingestion dose increase was halted or decreased according to the severity of allergic symptoms as needed. If moderate or severe allergic symptoms (eg, Grade 2 or 3 in Table S3) occurred, the ingestion dose was reduced until no symptom occurred with ingestion. We selected StU as the primary outcome in this study rather than the typical SU for cessation 4–8 weeks. Patient StU was evaluated via open-label OFC annually after a two-week avoidance of their corresponding allergy-causing foods. The OFC for StU evaluation was performed using the same protocol in both the 100%-dose group and 25%-dose group. These cumulative doses of OFC were equal to 1 heated whole egg, 100 mL of unheated milk, or 100 g of boiled udon noodle. These OFC doses were also the same as the maximum of 100%-dose OIT. They were divided into 1/8, 3/8, and 4/8 of the total dose per 30 min. Patients who developed symptoms during OFC in the first year were judged to have a desensitization status. They continued their OIT for another year with informed consent and underwent OFC again in the second year. However, the patients who passed OFC were deemed to have reached StU at 12 or 24 months. During the OIT, OFC for other food antigens was not restricted for patients with other food allergies.

After completing the study, the patients who reached StU with OIT were requested to consume their target dose of causative food (“a few minutes well-heated” scrambled eggs, raw milk, or boiled wheat noodles) at least once per week at home. They were also permitted to consume dairy products on other days to maintain their StU.^37^ They were followed-up continuously by an allergist to evaluate typical SU with longer avoidance, exercise,^38^ or deconditioned status (eg, tiredness, infection, and sickness).

### Study outcome

The primary endpoint was comparison of StU rates between the 100%- and 25%-dose groups in the first year. The secondary endpoints were: 1) StU rate at year 2 of follow-up; 2) immunological changes, which were measured every 6 months, in antigen-sIgE for egg-white, ovomucoid, milk, casein, wheat, and omega-5 gliadin, and antigen-sIgG and antigen-sIgG_4_ for egg-white, ovomucoid, casein, wheat, and omega-5 gliadin; and 3) rates of allergic symptoms and medication used during OIT ingestion for 2 years.

### Allergic reactions and treatments

Before beginning OIT, all patients received training for management of adverse allergic reactions at home (eg, when and how to use medication, including adrenaline self-injector). The ingestion, allergic reactions, and medications were recorded in a diary. The severity of allergic symptoms for patients was graded using modified anaphylactic symptom grading of the European Academy of Allergy and Clinical Immunology (EAACI),^39^ which could be easily modified using Table S3. The medications prescribed were used depending on the allergic reactions (eg, oral antihistamine, oral steroid, beta 2 agonist inhalation, or self-injectable adrenaline). All patients visited their respective hospital every 1–2 months for follow-up and treatment monitoring. They were also provided with telephone contact information for their hospital should allergic symptoms occur. In this study, only OIT food-related allergic symptoms were analyzed. Accidental adverse reactions outside of the OIT, caused by other foods, medications, and allergic complications (eg, rhinitis and asthma.) were excluded from the analysis.

### Measurement of IgE, IgG, and IgG_4_

In both groups, a blood test was performed every 6 months. The range of antigen-sIgE was 0.35–100 (kU_A_/L). The level of antigen-sIgE was measured using ImmunoCAP (Thermo Fisher Diagnostics K.K., Tokyo, Japan). Values ≤ 0.34 were calculated as 0.15 and those ≥ 100 as 101. The antigen-sIgG and antigen-sIgG_4_ were measured using a residual serum and human ELISA kit (Thermo Fisher Diagnostics K.K., Tokyo, Japan). The range of sIgG was 2.0–200.0, and sIgG_4_ was 0.07–30.0 (mg/L). Values ≤ 2.0 were calculated as 1.0 and those ≥ 200.0 as 201.0; meanwhile values ≤ 0.070 were calculated as 0.035 and ≥ 30.0 as 30.1.

### Statistics

We hypothesized that 100%-dose OIT would induce StU earlier than 25%-dose OIT. The assumed StU rates for the 100%- and 25%-doses were estimated to be 80% and 20%, respectively, which was calculated by the outcome of a preliminary outpatient OIT trial from 2009 to 2012 at the Department of Pediatrics, Sagamihara National Hospital (UMIN000011684, UMIN000011689). The required number of sample size for the 100%- and 25%-dose groups was calculated as 15 cases each for HE, CM, and wheat. ["Sampsi 0.8 0.2, p (0.8)”, power = 0.8, alpha = 0.05 (two-sided), dropout rate; 15%, using STATA 12, StataCorp LP, College Station, TX, USA].

According to the primary and secondary outcomes, comparisons of StU rates between the OIT 100%- and 25%-dose groups were conducted using Fisher's exact test. These outcomes were analyzed by intention-to-treat and per-protocol set analyses. We also defined the patients who discontinued OIT as allergic patients (worst-case scenario). The influence of dropout cases was analyzed by the tipping-point method.

Patient profile, age, antigen-sIgE level (for egg-white, milk, and wheat), and allergic symptom threshold were expressed as median values (interquartile range) and compared between the OIT groups using the Mann-Whitney *U* test. *p* < 0.05 was considered statistically significant. Allergic complications and the rate of past anaphylaxis or infantile eczema were analyzed using Pearson's chi-squared test.

Allergic symptoms and medication rates during OIT were also compared according to the other secondary outcomes using Pearson's chi-squared test. Changes in antigen-sIgE, -sIgG, and -sIgG_4_ during the follow-up period were compared using the Wilcoxon signed-rank test with SPSS 24 (IBM Corporation, Armonk, NY, USA). We made complete 100 datasets of antigen-sIgE, -sIgG, and -sIgG_4_ for the missing values through multiple imputation (linear regression).

## Results

### Patient characteristics

**Fig. 2:**
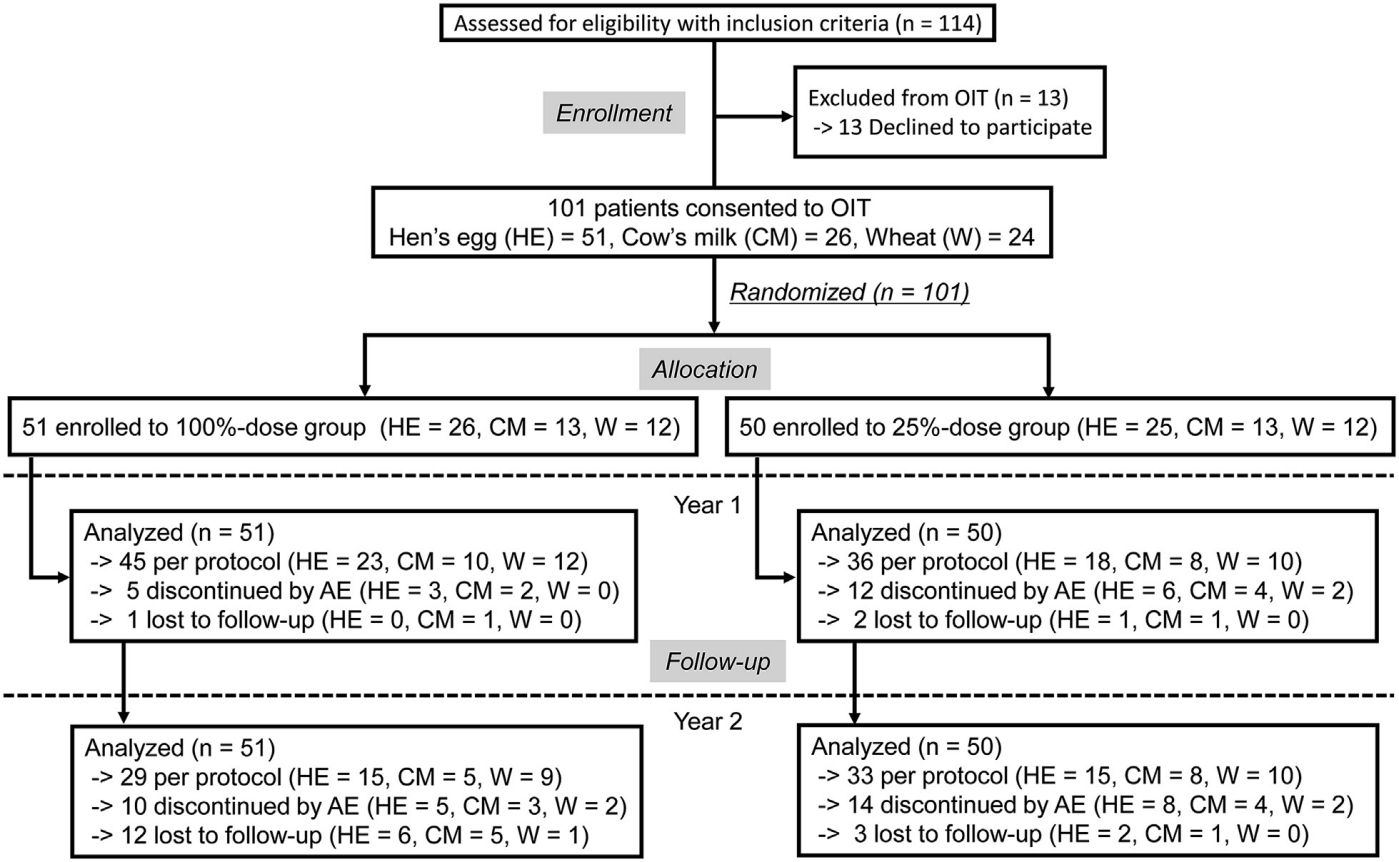
Patient diagram. OIT: oral immunotherapy, HE: hen's egg, CM: cow's milk, W: wheat. AE: adverse event. One-hundred and one patients who consented to OIT were randomized into two groups. OIT interruption occurred because of AE or loss to follow-up

A total of 101 patients consented to this study. The 51 patients in the 100%-dose group (HE = 26, CM = 13, wheat = 12), and the 50 in the 25%-dose group (HE = 25, CM = 13, wheat = 12) were examined by intention-to-treat analysis (Fig. 2). The age groups were from early childhood to middle school age, and their past history rates of anaphylaxis were approximately 30–69% (Table 1). The number of allergic symptoms and treatment at baseline OFC are expressed in Table S4. No statistically significant differences in baseline characteristics were observed between the groups. However, the numbers of patients assigned to the milk and wheat OIT were slightly lower than the estimated sample size, ie, up to 15 patients for each group.

**Table 1:** Patient profiles at baseline.

	HE 100%	HE 25%	CM 100%	CM 25%	W 100%	W 25%
Subjects, (n)	26	25	13	13	12	12
Age, (y)^*a*^	6.9 (4.7–9.2)	6.8 (5.2–8.9)	6.4 (6.0–7.7)	7.6 (6.1–10.4)	5.0 (3.7–5.5)	5.5 (4.5–5.8)
Gender, (M/F)	16/10	11/14	7/6	11/2	8/4	8/4
Past history of infantile eczema, (%)	50.0	60.0	38.5	46.2	75.0	75.0
BA, (%)	30.8	32.0	46.2	46.2	41.7	50.0
AD, (%)	53.8	64.0	61.5	53.8	75.0	66.7
AR, (%)	30.8	48.0	23.1	38.5	16.7	16.7
AC, (%)	15.4	12.0	7.7	23.1	8.3	0.0
Past history of An caused by HE, CM or W, (%)	30.8	56.0	69.2	38.5	58.3	50.0
Level of Ew-, M-, W-sIgE, (kU_A_/L)^*a*^	22.8 (8.50–30.0)	29.8 (16.1–56.4)	16.9 (7.47–47.2)	44.8 (15.4–68.8)	35.7 (13.3–56.7)	27.0 (7.76–52.4)
Allergic symptom threshold at baseline OFC, (protein, mg)^*a*^	1240 (388–1550)	1550 (581–1550)	510 (233–850)	425 (260–850)	351 (182–390)	338 (182–390)

OIT: oral immunotherapy, HE: hen's egg, CM: cow's milk, W: wheat, y: years, M: male, F: female, BA: bronchial asthma, AD: atopic dermatitis, AR: allergic rhinitis, AC: allergic conjunctivitis, An: anaphylaxis, Ew: egg-white, sIgE: specific immunoglobulin E, OFC: oral food challenge. Patient age, antigen-sIgE, and allergic symptom threshold are expressed as median values (interquartile range). Comparisons were made using Pearson's chi-squared test or the Mann-Whitney U test^*a*^. Regarding baseline patients' profiles, no statistically significant differences were observed for HE, CM or W allergy between the 100%- and 25%-dose groups (*p* > 0.05)

### Assessment of clinical response

**Fig. 3:**
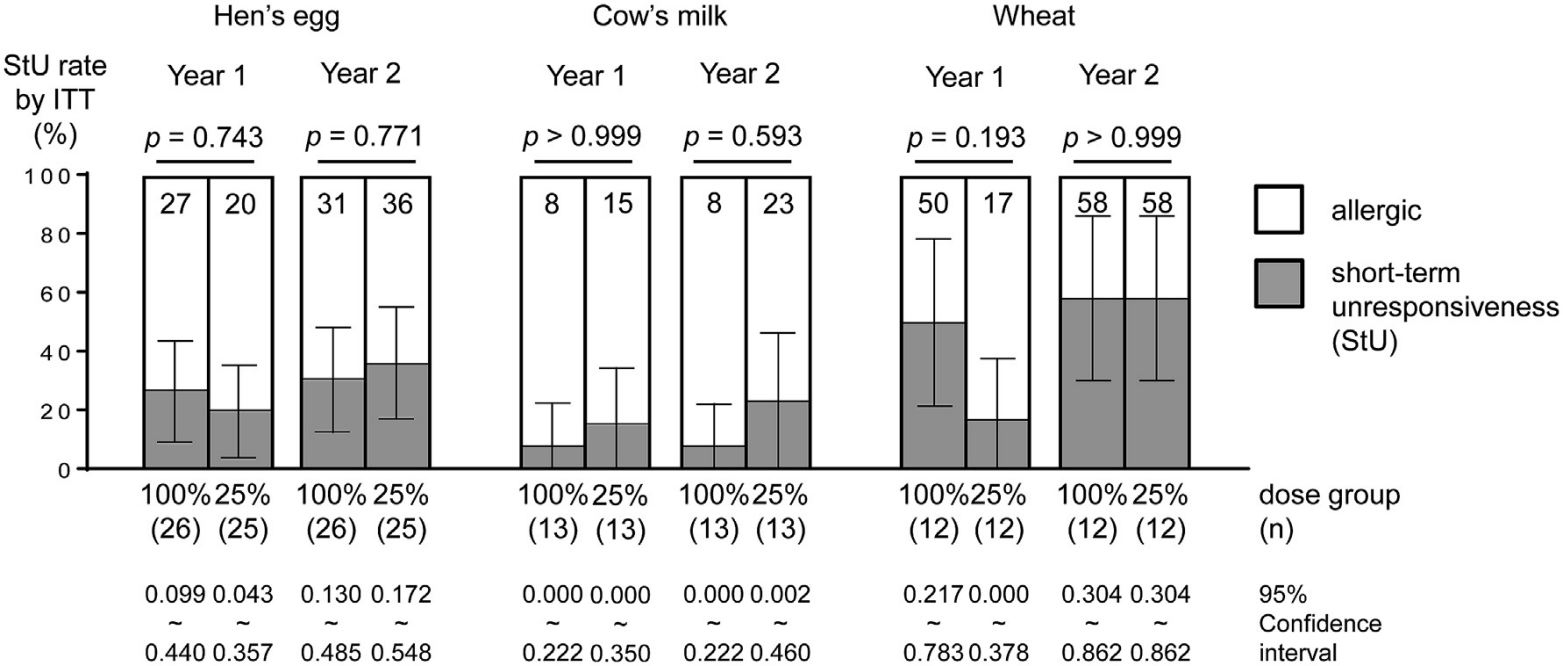
Comparison of outcome between the 100%- and 25%-dose groups. OIT: oral immunotherapy, StU: short-term unresponsiveness (gray bar), ITT: intention-to-treat analysis, allergic (white bar). StU was measured by an oral food challenge test after 2-week cessation of therapy. Y axis shows StU rate percentage. X axis shows the number of patients. StU rate (%) for (left) egg allergy after OIT follow-up per year; (middle) milk allergy after OIT; (right) wheat allergy after OIT. The StU rate was rounded off. Comparison of OIT outcome between the two groups was conducted by intention-to-treat analysis. *p* < 0.05 was considered statistically significant (Fisher's exact test). Confidence interval of population ratio is shown in the error-bar. Discontinued patients were considered allergic (worst-case scenario)

Two methods were employed to analyze the patients, namely intention-to-treat and per-protocol set analysis. Using the intention-to-treat analysis, all enrolled patients were analyzed and the StU rate (%) for year one in the 100%- and 25%-dose groups was 26.9 and 20.0 for HE, 7.7 and 15.4 for CM, and 50.0 and 16.7 for wheat (Fig. 3). The rates for year 2 were 30.8 and 36.0, 7.7 and 23.1, 58.3 and 58.3 for HE, CM, and wheat, respectively.

Using the per-protocol set analysis, only the patients who complied with the OIT protocol were analyzed, and the StU rate (%) for year one in the 100%- and 25%-dose groups was 30.4 and. 27.8 for HE, 10.0 and 25.0 for CM, and 50.0 and 20.0 for wheat (Fig. S1). The number of patients for desensitization and StU at year 1 are expressed in Table S5. The StU rates for year 2 were 53.3 and 60.0, 20.0 and 37.5, and 77.8 and 70.0 for HE, CM, and wheat, respectively.

There were no statistically significant differences in StU rates between the OIT for the 100%- and 25%-dose groups as evaluated by each antigen between year 1 and 2 by intention-to-treat and per-protocol analyses (*p* > 0.05). In addition, regarding the egg OIT outcome between year 1 and 2, the confidence interval distribution for their population ratio appeared to overlap. Regarding the milk OIT outcome, the confidence intervals did not overlap at years 1 and 2. However, regarding the wheat OIT outcome, the confidence intervals did not overlap at year 1, yet appeared to do so at year 2.

### Assessment of allergic reactions and treatments

The ratio of total adverse reactions per total ingestion times in the 25%-dose OIT group was significantly lower than that in the 100%-dose OIT group. Most of these allergic reactions during both OITs were mild, with the most frequent adverse reactions reportedly mild oral symptoms (Table 2). The frequency of gastrointestinal symptoms, including oral symptoms, during HE, CM, and wheat OIT was higher in the 100%-dose group than in the 25%-dose group. However, there was no obvious trend in the frequency of other organ-specific symptoms between these 2 groups.

**Table 2:** Comparison of allergic reactions and treatment between 100%-dose and 25%-dose OIT.

		HE 100%	HE 25%	CM 100%	CM 25%	W 100%	W 25%
Subjects, (n)		26	25	13	13	12	12
Total times of OIT ingestion, (n)		9546	9623	4990	5260	5032	6239
Ratio of total adverse reactions per total ingestion times, (%)		10.9	8.74^a^	15.4	10.3^a^	8.82	4.76^a^
Ratio of total treatments per total ingestion times, (%)		2.13	1.98	2.34	2.22	2.50	1.15^a^
Severity of symptoms, (%)	Grade 1 (mild)	10.2	7.57^a^	14.4	9.51^a^	7.63	3.77^a^
	Grade 2 (moderate)	0.60^a^	1.14	0.86	0.67	0.97	0.96
	Grade 3 (severe)	0.09	0.03	0.16	0.17	0.22	0.03^a^
Organ-specific symptoms, (%)	Skin	3.58	3.79	6.13	4.24^a^	3.66	3.51
	Gastrointestinal tract (oral symptom only)	7.85 (4.44)	5.67^a^ (3.22^a^)	10.1 (8.60)	6.16^a^ (5.10^a^)	3.60 (2.46)	0.50^a^ (0.32^a^)
	Respiratory tract	0.90^a^	1.63	2.97	2.70	2.44	1.49^a^
	Cardiovascular	0	0	0	0	0	0
	Neurological	0.18	0.25	1.12	0.25	0.12	0.06
Treatments at home, (%)	AH p.o.	2.10	1.92	1.94	2.17	2.34	1.14^a^
	Steroid p.o.	0.16^a^	0.31	0.24	0.25	0.12	0.10
	β2 agonist inhalation	0.05^a^	0.30	0.78	0.40^a^	0.42	0.05^a^
	Adrenaline i.m.	0	0	0.02	0.06	0	0.02
Treatments at hospital, (%)	Emergency visit	0.08	0.06	0.04^a^	0.23	0.02	0.08
	AH i.v. or i.m.	0	0	0	0	0	0.02
	Steroid i.v.	0.02	0	0.02	0.02	0	0.03

OIT: oral immunotherapy, HE: hen's egg, CM: cow's milk, W: wheat, AH: antihistamine, p.o.: per oral, i.v.: intravascular, i.m.: intramuscular. Frequency of severity of symptoms, organ-specific symptoms and treatments were calculated by number per total ingestion. Comparisons of HE, CM and W allergy between 100%-dose and 25%-dose OIT were conducted using Pearson's chi-squared test

a *p* < 0.05 was considered statistically significant lower.

Regarding the ratio of total treatments per total ingestion during OIT, the rate in the 25%-dose OIT group was significantly lower than that in the 100%-dose OIT group for wheat only. However, no obvious trend was observed in the frequency of using any medications between these two groups.

There were 5 cases of intramuscular adrenaline injections used during OIT (CM in the 100%-dose group = 1, CM in the 25%-dose group = 3, wheat in the 25%-dose group = 1), and 3 out of 5 cases were defined as anaphylaxis: Grade 4 or 5 according to the modified WAO grading system^40^ (CM in the 100%-dose group = 1, CM in the 25%-dose group = 1, wheat in the 25%-dose group = 1). Only one case of CM in the 25%-dose group had reduced consciousness and was classified as Grade 5 according to the grading system of the World Allergy Organization (WAO). However, no cases of anaphylactic shock, respiratory failure or hypotension were observed. Importantly, the 3 anaphylactic cases and 1 non-anaphylactic case out of 5, did not maintain the 1 h rest rule after OIT ingestion. The remaining 1 non-anaphylactic case developed acute enteritis before ingestion. Furthermore, another patient treated with adrenaline injection due to accidental ingestion, was not related to OIT ingestion (HE = 1). However, this case's injection was excluded from the analysis of this study.

### Immunological changes

**Fig. 4:**
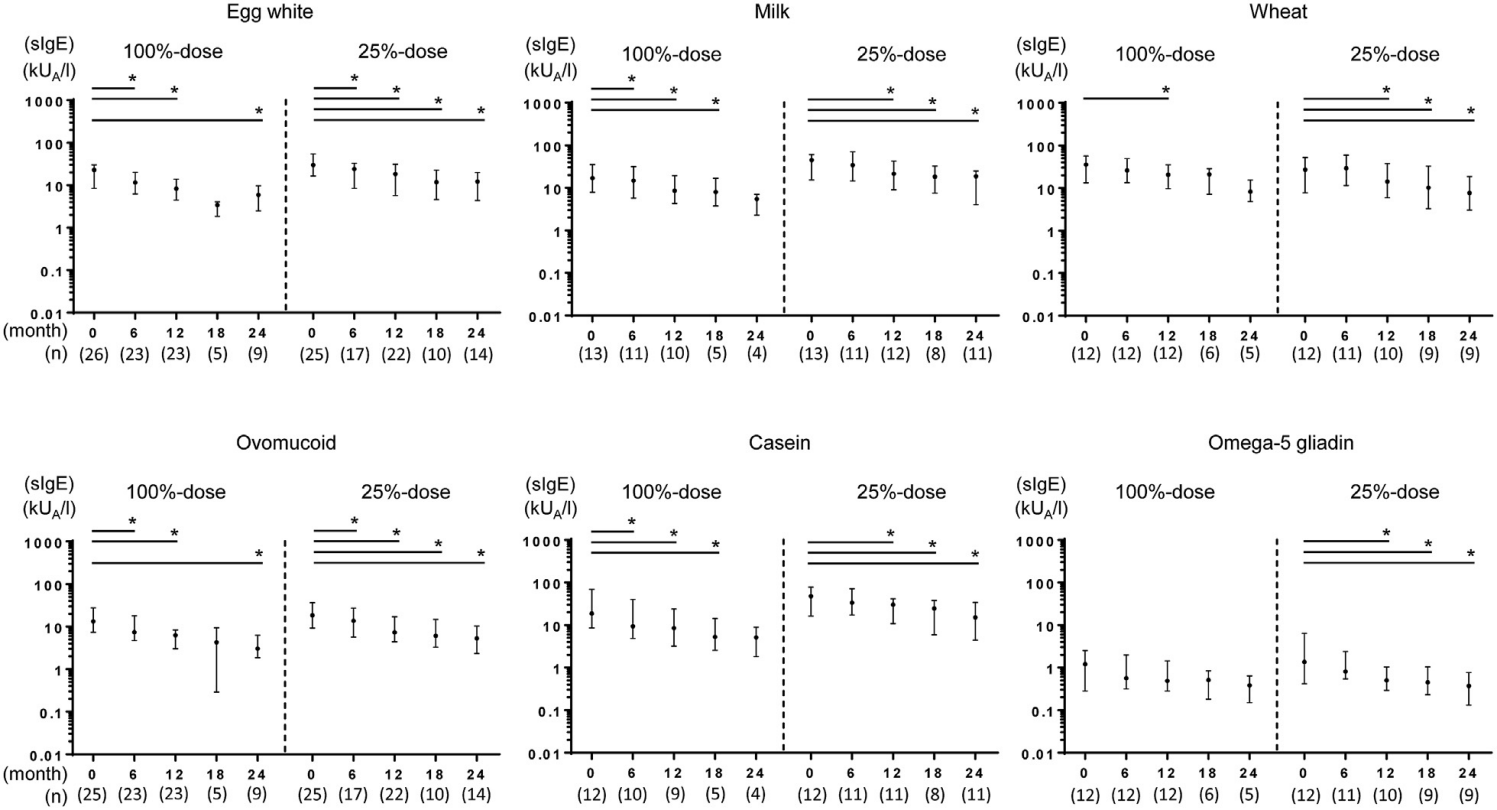
Immunological changes in sIgE during OIT. OIT: oral immunotherapy, sIgE: specific immunoglobulin E. Antigen-sIgE (kU_A_/L) levels for Left: egg-white (top) and ovomucoid (bottom) by each group during OIT, measured each 6 months; Middle: milk (top) and casein (bottom); Right: wheat (top) and omega-5 gliadin (bottom). Data are expressed as median values (interquartile range). *p* < 0.05 was considered statistically significant (horizontal line and ∗). Comparisons were conducted using the Wilcoxon signed-rank test (baseline; 0 month)

The levels of antigen-sIgE for egg-white, ovomucoid, milk, casein, and wheat decreased significantly in the 100%- and 25%-dose groups at 12 months, compared to those at baseline (Fig. 4). However, we did not observe a significant association between the StU status and the reduction of antigen-sIgE levels. With regards to the omega-5 gliadin levels at 12 months, this was significantly decreased in the 25%-dose group only. The levels of antigen-sIgE for egg-white, ovomucoid, milk, casein, wheat, and omega-5 gliadin in the 25%-dose group also significantly decreased at 24 months. However, in the 100%-dose group, the levels of antigen-sIgE for only egg-white and ovomucoid significantly decreased at 24 months. Furthermore, due to insufficient sample size, we could not detect obvious changes in antigen-sIgG and -sIgG_4_ levels in the 100%- and 25%-dose groups (Fig. S2 and S3). Integrated *p* values of immunological changes by multiple imputation for missing values are shown in Table S6.

### Discontinuation of OIT

A total of 22 and 17 patients discontinued OIT in the 100%-dose group and 25%-dose group by the 2-year follow-up, respectively. Moreover, 10 out of 22 patients in the 100%-dose group, and another 14 out of 17 patients in the 25%-dose group discontinued their OIT owing to adverse events (Fig. 2). The reasons for discontinuing OIT for the 10 and 14 patients in the 100%- and 25%-dose groups were allergic symptoms (n = 4 vs. 4), children disliking the food (n = 0 vs. 5), sudden termination of hospital visits (n = 6 vs. 2), enrolling in a different OIT study (n = 0 vs. 1), worsening of atopic dermatitis (n = 0 vs. 1), and parental anxiety (n = 0 vs. 1), respectively (Table S7).

Although we extended the original research period, 4 out of 9 hospitals chose not to extend the study participation period from 12 to 24 months. Hence, the patients in these 4 hospitals were lost to follow-up at year 2, regardless of whether their OIT could induce desensitization. Therefore, 11 patients in the 100%-dose group and one patient in the 25%-dose group stopped their follow-up at the first year. Other reasons for loss to follow-up were moving away (n = 1 vs. 0), acute infection (n = 0 vs. 1), and mother's pregnancy (n = 0 vs. 1).

## Discussion

The efficacy of OIT itself has been evaluated for HE, CM, and peanut allergy in previous randomized control trials^3^, ^4^, ^5^, ^6^^,^^8^^,^^9^^,^^15^^,^^17^, ^18^, ^19^, ^20^^,^^22^^,^^23^^,^^25^^,^^26^^,^^33^^,^^34^ and systematic reviews.^41^, ^42^, ^43^, ^44^, ^45^, ^46^ In addition, a five-day medium dose (2808 mg) of rush OIT in persistent HE allergic children was recently found to desensitize 94% of allergic patients with moderate to mild adverse effects.^47^ Meanwhile, low dose OIT seems to improve the safety of allergen ingestion for severe allergic patients.^22^^,^^29^, ^30^, ^31^ Thus, the adopted protocols, study designs, and endpoints are often diverse, making it difficult to compare their efficacies in terms of dose-dependent outcome.^48^

In 2017, Vickery et al reported the similar effectiveness of peanut OIT with the comparison of 10 times maintenance doses.^33^ In other previous OIT reports for milk^32^ and wheat,^34^ no significant differences in outcomes were observed when compared to twice the maintenance dose. However, a potential interference may occur with combined SLIT for milk OIT,^32^ or it may be difficult to achieve SU in highly allergic patients.^34^ In addition, the optimal ingestion dose, particularly for HE allergen during OIT, was uncertain. To the best of our knowledge, our preliminary, proof of concept study is the first report on the dose-dependent OIT outcomes of HE allergic patients. Our data may prove helpful to inform the design of similar studies in larger populations. Furthermore, the severity of allergy in the patients of this study was moderate, with severe anaphylactic patients being excluded to ensure clear dose-differences in outcome. Moreover, our definition of low dose was set lower than in past reports;^32^^,^^34^ and no previous study has described a comparison of more than 4 times larger dose-differences in CM and wheat OIT outcomes using such a patient population and focusing on the lower dose.

### Dose-dependent outcome

In this study, a certain dose-dependent outcome was not identified during HE, CM, and wheat OIT. The effects of 25%-dose egg OIT might not be inferior to those of the 100%-dose in terms of the StU rate after a 2-year clinical follow-up. On the contrary, the StU rates of milk OIT were lower than that of egg and wheat, even at the 100%-dose. Longer follow-up periods may be required to compare dose dependency for milk OIT. Regarding wheat OIT, the 100%-dose seemed to induce StU at only year 1, which is earlier than that induced by the 25%-dose. However, this difference became negligible after the 2-year follow-up. Our findings did not, therefore, indicate that similar efficacy is obtained with a low and target dose OIT for milk and wheat. To examine the influence of dropout cases, tipping-point analysis was conducted, and the results are shown in Fig. S4. There was less influence of dropout cases on the primary outcome at year 1. In addition, it was difficult to compare our outcome to that of past OIT studies as we excluded patients who had been treated with adrenaline injection at the baseline OFC for safety. There might also be a slight placebo bias owing to the open-labeled nature of this study.

### Feasibility of 25%-dose OIT

Although it may be easier for children to ingest low doses of OIT, particularly peanut,^49^ continuation of HE, CM, and wheat OIT for children is nevertheless stress-laden if the therapy has to be continued over a long period, even if the maintenance dose is as small as 25% of OIT. To further reduce stress during OIT, very low doses may be required, as used in previous studies.^22^^,^^29^, ^30^, ^31^ This observation implies the need for careful consideration of indication for OIT, maintenance dose, and treatment period for this class of patients. Such considerations may, however, change in the future with the emergence of individualized therapy that can stratify patients by the severity of their allergies to provide enhanced treatment safety, convenience, and efficacy.^50^

### Adverse reactions of lower dose OIT

In this study, 49 out of 101 (48.5%) OIT patients had a past history of anaphylaxis, although most adverse allergic symptoms that occurred during OIT were mild. In addition, the rate of total allergic symptoms in the 25%-dose group was lower than that in the 100%-dose group, apart from the adrenaline injection frequency. Our results (25%-dose OIT) and those of Vickery et al.^33^ (10%-dose OIT) suggest that low dosages might decrease the rate of adverse allergic symptoms. However, 50%-dose OIT^32^^,^^34^ was unable to significantly decrease the adverse symptom rate. Thus, to meaningfully decrease adverse symptoms, a low dose OIT of at least ≤25% may be necessary.

Moreover, several cases of adrenaline injections occurred during milk and wheat OIT. The co-factors of anaphylaxis were exercising, going out, or bathing within 1 h of OIT ingestion. Although the patients were advised on the “rest rule” during OIT, some parents became careless, suggesting the need to provide more comprehensive explanations to patients and their legal guardians in relation to the importance of strictly adhering to the rules/guidelines associated with OIT, especially for milk and wheat OIT. Regardless, the 25%-dose OIT significantly reduced the mild allergic symptoms. However, it was difficult to conclude whether the safety improved in the 25% group compared to the 100% group, since four adrenaline injections for milk and wheat reactions were administered in the 25% group. Further reduction of the maintenance dose may be necessary to reduce the risk of moderate or severe symptoms caused by milk and wheat OIT.

### Immunological changes

In this study, low doses of OIT induced certain reduction in the levels of antigen-sIgE against their causative antigens at 12 months. Moreover, the 25%-maintenance dose appeared to induce similar levels of causative antigen-sIgE at 12 months as that induced by the larger doses, as supported by immunological changes. However, antigen-sIgG and -sIgG_4_ could be measured only in few cases owing to insufficient residual serum.

### Limitations

There are a number of limitations noted in the current study. First, most traditional OIT studies measured typical SU up to 4–10 weeks after cessation of therapy. However, in this study, the primary outcome was StU, which was only measured at 2 weeks after cessation of therapy. This two-week avoidance was used to evaluate clinical efficacy of OIT in previous reports.^11^^,^^12^^,^^14^^,^^29^, ^30^, ^31^ However, our StU definition was unlikely to distinguish desensitization from typical SU. If the cessation period was longer, different results for OIT outcome might have been obtained. However, in terms of efficacy comparison between the 100%-dose and 25%-dose OIT, we considered that StU might have slightly affected the primary endpoint. Moreover, HE, CM, and wheat could be eaten regularly as diet, and thus 2 weeks of cessation was clinically appropriate to suspend ingestion owing to acute infection, travel, or other reasons during OIT.

Second, the follow-up period of this study was extended from 1 year to 2 years (trial registry was fixed at February 2014), and owing to the lack of case follow-up, or residual serum at year 2, there were some missing values of immunological changes, particularly for the antigens specific for IgG and IgG_4_ in the OIT group.

In addition, we aimed to investigate the efficacy of OIT compared to the natural course without therapy in terms of the secondary outcome. However, there were not enough patients in the control group for valid statistical analysis, as many preferred to participate in the trial in the OIT groups. Moreover, it was difficult to keep patients in the control group for 1–2 years, as they all had national insurance coverage and were able to choose other allergists freely and for a low price in Japan. Only 6 patients (HE = 3, CM = 2, wheat = 1) who did not consent to OIT were enrolled in the control group, none of whom attained tolerance for the StU target dose during the 2-year follow-up. Therefore, we could not compare OIT efficacy to the natural outgrowing tolerance.

## Conclusions

This investigation was unable to show a statistically significant difference between the 100%-dose group and the reduced 25%-dose group. Owing to insufficient statistical power, particularly for milk and wheat, follow-up studies using a larger study population will be required to replicate and validate our finding that reducing the maintenance dose of OIT may exert equivalent therapeutic efficacy as that achieved by the target dose of OIT. However, limited to the results on egg OIT, this study may have suggested that the 25%-dose was similar to the 100%-dose in terms of clinical effectiveness after 2 years of follow-up. Reducing the maintenance dose for eggs, milk, and wheat may effectively lower the symptoms associated with their consumption compared to the target OIT dose. However, the 25%-dose group for milk and wheat required a few adrenaline injections. Further reduction of the maintenance dose may be necessary to reduce the risk of anaphylaxis, particularly for the milk and wheat OIT. Furthermore, patient compliance, stress inflicted by eating, and quality of life associated with StU needs to be compared among the 100%-, 25%-, and further reduction-dose OIT.

## Author contributions

Kiyotake Ogura managed this research, analyzed the clinical data, and wrote this manuscript. Takanori Imai, Komei Ito, Naoyuki Kando, Masanori Ikeda, Rumiko Shibata, Yoko Murakami, Takao Fujisawa, Mizuho Nagao, Norio Kawamoto, Naomi Kondo, Atsuo Urisu, Ikuya Tsuge, Yasuto Kondo, Kazuko Sugai, Osamu Uchida contributed to brush up this study and to manage the patients in each hospital. Noriyuki Yanagida, Sakura Sato, Mitsuyoshi Urashima, Masami Taniguchi, and Motohiro Ebisawa contributed to create this clinical research, advise statistically, and teach this manuscript.

## Potential competing interests

The authors report no competing interests.

## Funding

This work was supported by the 10.13039/501100003478Ministry of Health, Labour and Welfare of Japan [grant number 201414009A].

## Role of the funding source

The funding source had no role in study design, data collection, analysis. There was no interpretation of data, writing of the report and in the decision to submit the article for publication.

## Submission declaration

This study has not been published before in any journal. However, a part of data was showed in International Pediatric Allergy Symposia at the 52nd and 53rd annual meeting of Japanese Society of Pediatric Allergy and Clinical Immunology in 2015 and 2016. These abstracts were showed on the Japanese journal of Pediatric Allergy and Clinical Immunology.

## Ethical considerations

All patients and their legal guardians provided consent for participation in this study, which was approved by the ethics committees of our hospital (Rinri-2012121112) and all the participating hospitals. The study also conformed to the ethical principles of the 2013 Declaration of Helsinki as well as the ethical guidelines for Medical and Health Research Involving Human Subjects in Japan. Authorship was limited to have a certain contribution to this study. All human serums were handled in compliance with standard precautions in each hospital.

## Consent for publication

All authors consented for publication in this study. The authors do not publish this same research in other articles or journals.

## Clinical trial registration

The trial registration number was UMIN000009373. https://upload.umin.ac.jp/cgi-open-bin/ctr_e/ctr_view.cgi?recptno=R000011019.

## Availability of data and materials

The anonymized datasets of this study are available in the UMIN-ICDR repository, with privacy protection. https://upload.umin.ac.jp/cgi-bin/icdr/ctr_file_dl.cgi?recptno=R000011019&file_id=00001&file_kind=3.

## Declaration of competing interest

The authors have no conflict of interest to disclose with respect to this study.

## Appendix A Supplementary data

**Supplemental Table S1**: Examples of total challenge doses in OFC. OFC: oral food challenge. All doses are expressed as protein dose of causative food (mg), which was modified using Japanese guidelines for food allergy 2017. The full dose refers to a dose to confirm the tolerance acquisition, determined from a single meal quantity in elementary-school pupils.

**Supplemental Table S2**: Up-dosing schedule of the OIT group. OIT: oral immunotherapy, ^*a*^whole egg protein, HE: hen’s egg, CM: cow’s milk. All doses are expressed as protein dose of causative food (mg). The ingestion doses of OIT were gradually increased to a maintenance dose, if the allergic symptoms did not occur for more than five consecutive days. A total of 20 dose levels were assigned for egg, and 25 for milk and wheat. A minimum of 100 days for egg, and 125 days for milk and wheat would be required to achieve the maintenance dose.

**Supplemental Table S3**. Grading of symptoms. Hypotension was defined as systolic blood pressure of < 70 mm Hg (ages 1 month to < 1 year), < (70 mm Hg + [2 × age]) (ages, 1–10 years), and < 90 mm Hg (> 10 years). Mild hypotension was defined as systolic blood pressure of < 80 mm Hg (ages 1 month to < 1 year), < (80 mm Hg + [2 × age]) (ages, 1–10 years), and < 100 mm Hg (> 10 years).

**Supplemental Table S4**. Patient allergic symptoms and treatment at baseline OFC. OFC: oral food challenge, HE: hen’s egg, CM: cow’s milk, p.o.: per oral, i.v.: intravascular, i.m.: intramuscular. The number of patients for allergic symptoms and treatment at baseline OFC were expressed by the each OIT group. ^*a*^There were some overlaps. The severity of allergic symptoms for patients was graded using Table S3. Comparisons were made using Pearson's chi-squared test. No statistically significant differences were observed for HE, CM or wheat allergy between the 100% and 25%-dose groups (*p* > 0.05).

**Supplemental Table S5**: The number of patients for desensitization and StU at year 1. HE: hen’s egg, CM: cow’s milk, StU: short-term unresponsiveness. The number of patients for desensitization and StU at year 1 were expressed by the each OIT group. The maintenance doses for desensitization were different between 100%-OIT group and 25%-OIT group, according to the study protocol. StU was measured by passing an oral food challenge test after 2-weeks cessation of therapy. The target doses for StU at the oral food challenge test were same in the 100%-OIT group and the 25%-OIT group, for each food.

**Supplemental Table S6**: Integrated *p* values of immunological changes by multiple imputation for missing values. *p*: *p* value, sIgE: specific immunoglobulin E, sIgG: specific immunoglobulin G, sIgG_4_: specific immunoglobulin G_4_. Regarding the missing values, complete data sets were made 100 times by multiple imputation. Comparisons of immunological changes were made using the Wilcoxon signed-rank test compared to baseline (0 month). Integrated *p* value was shown as mean (standard error) which was calculated by using all imputed data sets. *p* < 0.05 was considered statistically significant (gray area).

**Supplemental Table S7**: Reasons for OIT discontinuation. HE: hen’s egg, CM: cow’s milk, OIT: oral immunotherapy. The number of patients who discontinued OIT and left their OIT group are expressed within each OIT group, respectively.

**Supplemental Fig. S1**: Comparison of outcome between 100%-dose group and 25%-dose group. OIT: oral immunotherapy, StU: short-term unresponsiveness (gray bar), PPS: per-protocol set analysis, allergic (white bar). StU was measured by an oral food challenge test after 2-week cessation of therapy. Y axis represents StU rate percentage. X axis represents the number of patients. StU rate (%) for (left) egg allergy after OIT follow-up per year; (middle) milk allergy after OIT; (right) wheat allergy after OIT. The StU rate was rounded off. The comparison of OIT outcome between two groups was evaluated using the per-protocol set analysis. *p* < 0.05 was considered statistically significant (Fisher’s exact test). Confidence interval of population ratio were shown in the error-bar. Discontinued patients were considered as allergic (worst-case scenario).

**Supplemental Fig. S2**: Immunological changes of sIgG during OIT. OIT: oral immunotherapy, sIgG: specific immunoglobulin G. Antigen-sIgG (mg/l) levels for Left: egg-white (top) and ovomucoid (bottom) by each group during OIT, measured every 6 months; Middle: casein (bottom); Right: wheat (top) and omega-5 gliadin (bottom). Data are expressed as median values (interquartile range). *p* < 0.05 was considered statistically significant (horizontal line and ∗). Comparisons were made using the Wilcoxon signed-rank test. (baseline; 0 month).

**Supplemental Fig. S3**: Immunological changes of sIgG_4_ during OIT. OIT: oral immunotherapy, sIgG_4_: specific immunoglobulin G_4_. Antigen sIgG_4_ (mg/l) levels for Left: egg-white (top) and ovomucoid (bottom) by each group during OIT, measured every 6 months; Middle: casein (bottom); Right: wheat (top) and omega-5 gliadin (bottom). Data are expressed as median values (interquartile range). *p* < 0.05 was considered statistically significant (horizontal line and ∗). Comparisons were made using the Wilcoxon signed-rank test (baseline; 0 month).

**Supplemental Fig. S4**: Tipping-point analysis of outcome between 100% and 25% dose groups in years 1 and 2. X- and Y-axes represent the number of stopped OIT (due to discontinuation and loss to follow-up) patients, who were considered as “allergic” in the 100% and 25% dose groups, respectively. Intention-to-treat analysis was used for the comparison. *p* < 0.05 (gray area) was considered significant (Fisher’s exact test). Worst-case scenario: all patients in both groups were allergic; best-case scenario: all patients in both groups were short-term unresponsiveness. OIT, oral immunotherapy.
