# Supplementary material for: Evaluation of oral immunotherapy efficacy and safety by maintenance dose dependency: A multicenter randomized study
Source: World Allergy Organ J. 2020 Sep 29;13(10):100463. doi: 10.1016/j.waojou.2020.100463 (PMC7527748; doi:10.1016/j.waojou.2020.100463)
Supplement: Multimedia component 1 [file mmc1.pdf]

Supplemental Tables

Table S1. Examples of total challenge doses in OFC

Doses (protein, mg)	Hen’s egg	Cow’s milk	Wheat
**Low dose**	194	102	50–75
**Medium dose**	775–3100	510–170	375–1250
**Full dose**	6200	6800	5000

Table S2. Up-dosing schedule of the OIT group

Dose (protein, mg)	HE[a] 100%	HE[a] 25%	CM 100%	CM 25%	Wheat 100%	Wheat 25%
**1**	124	124	10	10	8	8
**2**	155	155	14	14	10	10
**3**	186	186	17	17	13	13
**4**	248	217	20	20	16	16
**5**	310	248	27	24	21	18
**6**	372	279	34	27	26	21
**7**	496	310	51	34	39	26
**8**	620	341	68	41	52	31
**9**	744	372	85	48	65	36
**10**	930	434	102	54	78	42
**11**	1240	496	136	61	104	47
**12**	1550	558	170	68	130	52
**13**	1860	620	204	85	156	65
**14**	2170	744	272	102	208	78
**15**	2480	868	340	136	260	104
**16**	3100	992	408	170	312	130
**17**	3720	1116	510	204	390	156
**18**	4340	1240	680	238	520	182
**19**	5270	1364	850	272	650	208

**20**	6200	1550	1020	340	780	260
**21**	-	-	1360	408	1040	312
**22**	-	-	1700	476	1300	364
**23**	-	-	2210	578	1690	442
**24**	-	-	2720	680	2080	520
**25**	-	-	3400	850	2600	650

Table S3. Grading of symptoms

Organ	Grade 1 (mild)	2 (moderate)	3 (severe)
Skin	Localized urticaria, exanthema, wheal, pruritus	Generalized urticaria, exanthema, wheal, pruritus	
	Swollen lip or eyelid	Swollen face	
Gastrointestinal tract	Pruritus of the throat or oral cavity	Throat pain	
	Mild abdominal pain	Moderate abdominal pain	Cramps
	Nausea, emesis, diarrhea	Recurrent emesis, diarrhea	Continuous emesis, loss of bowel control
Respiratory tract	Intermittent cough, nasal congestion, sneezing, rhinorrhea	Repetitive cough	Persistent cough, hoarseness, "barky" cough

		Chest tightness, wheezing detectable via auscultation	Audible wheezing, dyspnea, cyanosis, saturation < 92%, swallowing or speaking difficulties, throat tightness, respiratory arrest
Cardiovascular		Pale face, mild hypotension, tachycardia (increase > 15 beats/min)	Hypotension, dysrhythmia, severe bradycardia, cardiac arrest
Neurological	Change in activity level, tiredness	“Light-headedness,” feeling of “pending doom,” somnolence, headache	Confusion, loss of consciousness, incontinence

Table S4. Patient allergic symptoms and treatment at baseline OFC

**Symptoms and treatment**		**HE 100%**	**HE 25%**	**CM 100%**	**CM 25%**	**Wheat 100%**	**Wheat 25%**
Skin	1 (mild)	13	10	8	3	5	4
	2 (moderate)	6	4	2	5	5	7
Gastrointestinal tract	1 (mild)	7	1	2	1	2	3
	2 (moderate)	4	6	5	7	8	7
	3 (severe)	1	1	3	0	1	0
Respiratory tract	1 (mild)	9	8	4	7	0	1
	2 (moderate)	13	15	1	0	1	1
	3 (severe)	2	0	0	0	0	0
Cardiovascular or Neurological	1 (mild)	7	5	1	2	0	1
	2 (moderate)	1	1	0	0	1	0
	3 (severe)	0	0	0	0	0	0
Antihistamine[a]	p.o.	13	11	6	9	7	11
	i.v. or i.m.	5	8	1	3	2	2
Steroid[a]	p.o.	4	6	0	2	0	1
	i.v.	3	5	0	1	1	1
β2 agonist	inhalation	4	7	7	6	8	9
Adrenaline	i.m.	0	0	0	0	0	0

Table S5. The number of patients for desensitization and StU at year 1

Food	Status	100%-OIT
**HE**	Desensitization to 6200 mg (whole egg protein)	12
	StU to 6200mg (whole egg protein)	7
**CM**	Desensitization to 3400 mg (milk protein)	7
	StU to 3400mg (milk protein)	1
**Wheat**	Desensitization to 2600 mg (wheat protein)	4
	StU to 2600 mg (wheat protein)	6

Food	Status	25%-OIT
**HE**	Desensitization to 1550 mg (whole egg protein)	11
	StU to 6200mg (whole egg protein)	5
**CM**	Desensitization to 850 mg (milk protein)	4
	StU to 3400mg (milk protein)	2
**Wheat**	Desensitization to 650 mg (wheat protein)	8
	StU to 2600 mg (wheat protein)	2

Table S6. Integrated *p* values of immunological changes by multiple imputation for missing values

**Antigen sIgE**		**Egg-white**	**Ovomucoid**	**Milk**	**Casein**	**Wheat**	**Omega-5 gliadin**
**100%-dose group**	0-6 months	$p < 0.001$ (< 0.001)	$p < 0.001$ (< 0.001)	$p = 0.047$ (0.003)	$p = 0.028$ (0.003)	$p = 0.266$ (< 0.001)	$p = 0.105$ (< 0.001)
	0-12 months	$p < 0.001$ (< 0.001)	$p < 0.001$ (< 0.001)	$p < 0.001$ (< 0.001)	$p = 0.003$ (< 0.001)	$p = 0.027$ (< 0.001)	$p = 0.064$ (< 0.001)
	0-18 months	$p = 0.063$ (< 0.001)	$p = 0.125$ (< 0.001)	$p = 0.007$ (0.064)	$p < 0.001$ (< 0.001)	$p = 0.065$ (0.017)	$p = 0.124$ (0.013)
	0-24 months	$p < 0.001$ (< 0.001)	$p < 0.001$ (< 0.001)	$p = 0.125$ (< 0.001)	$p = 0.125$ (< 0.001)	$p = 0.063$ (< 0.001)	$p = 0.063$ (< 0.001)
**25%-dose group**	0-6 months	$p = 0.008$ (0.002)	$p = 0.003$ (< 0.001)	$p = 0.238$ (0.019)	$p = 0.102$ (0.008)	$p = 0.791$ (0.009)	$p = 0.315$ (0.017)
	0-12 months	$p = 0.001$ (< 0.001)	$p < 0.001$ (< 0.001)	$p = 0.004$ (< 0.001)	$p = 0.005$ (< 0.001)	$p = 0.002$ (< 0.001)	$p = 0.001$ (< 0.001)
	0-18 months	$p < 0.001$ (< 0.001)	$p = 0.001$ (< 0.001)	$p = 0.010$ (0.033)	$p = 0.006$ (< 0.001)	$p = 0.002$ (< 0.001)	$p = 0.002$ (< 0.001)
	0-24 months	$p < 0.001$ (< 0.001)	$p < 0.001$ (< 0.001)	$p = 0.003$ (< 0.001)	$p = 0.002$ (< 0.001)	$p = 0.002$ (< 0.001)	$p < 0.001$ (< 0.001)

**Antigen sIgG**		**Egg-white**	**Ovomucoid**		**Casein**	**Wheat**	**Omega-5 gliadin**

**100%-dose group**	0-6 months	p = 0.193 (0.018)	p = 0.026 (0.004)	p = 0.125 (< 0.001)	p = 0.904 (0.011)	p = 0.754 (0.019)
	0-12 months	p = 0.052 (0.008)	p = 0.054 (0.008)	p = 0.625 (< 0.001)	p = 0.269 (0.017)	p = 0.303 (0.026)
	0-18 months	p = 0.027 (0.005)	p = 0.011 (0.002)	p = 0.250 (< 0.001)	p = 0.382 (0.015)	p = 0.231 (0.016)
	0-24 months	p = 0.193 (0.014)	p = 0.124 (0.010)	p = 0.625 (< 0.001)	p = 0.862 (0.013)	p = 0.055 (0.003)
**25%-dose group**	0-6 months	p = 0.120 (0.016)	p = 0.090 (0.014)	p = 0.455 (0.025)	p = 0.012 (< 0.001)	p = 0.234 (< 0.001)
	0-12 months	p = 0.096 (0.009)	p = 0.013 (0.001)	p = 0.200 (0.020)	p = 0.701 (0.029)	p = 0.239 (0.027)
	0-18 months	p = 0.324 (0.028)	p = 0.244 (0.025)	p = 0.700 (0.023)	p = 0.508 (0.014)	p = 0.715 (0.015)
	0-24 months	p = 0.300 (0.025)	p = 0.096 (0.011)	p = 0.388 (0.020)	p = 0.614 (0.032)	p = 0.362 (0.022)

**Antigen sIgG₄**		**Egg-white**	**Ovomucoid**	**Casein**	**Wheat**	**Omega-5 gliadin**
**100%-dose group**	0-6 months	p = 0.280 (0.020)	p = 0.063 (< 0.001)	p = 0.250 (< 0.001)	p = 0.549 (0.019)	p = 0.554 (0.030)
	0-12 months	p = 0.025 (0.005)	p = 0.031 (< 0.001)	p = 0.125 (< 0.001)	p = 0.867 (0.018)	p = 0.833 (0.012)

	0-18 months	$p = 0.009$ (0.001)	$p = 0.250$ (< 0.001)	$p = 0.128$ (0.003)	$p = 0.551$ (0.025)	$p = 0.577$ (0.026)
	0-24 months	$p = 0.072$ (0.007)	$p = 0.094$ (< 0.001)	$p > 0.999$ (< 0.001)	$p = 0.702$ (0.024)	$p = 0.615$ (0.028)
**25%-dose group**	0-6 months	$p = 0.164$ (0.019)	$p = 0.089$ (0.013)	$p = 0.442$ (0.016)	$p = 0.301$ (< 0.001)	$p = 0.688$ (< 0.001)
	0-12 months	$p = 0.042$ (0.005)	$p = 0.003$ (< 0.001)	$p = 0.544$ (0.022)	$p = 0.708$ (0.020)	$p = 0.360$ (0.033)
	0-18 months	$p = 0.248$ (0.027)	$p = 0.282$ (0.031)	$p = 0.374$ (0.017)	$p = 0.046$ (0.005)	$p = 0.526$ (0.030)
	0-24 months	$p = 0.093$ (0.013)	$p = 0.016$ (0.003)	$p = 0.656$ (0.018)	$p = 0.105$ (0.007)	$p = 0.243$ (0.019)

Table S7. Reasons for OIT discontinuation

Reason	HE	CM	Wheat
Discontinuation by adverse events			
Allergic symptoms (anaphylaxis)		n = 1 (100%-OIT)	
		n = 1 (25%-OIT)	
Allergic symptoms (not anaphylaxis)	n = 2 (100%-OIT)	n = 1 (100%-OIT)	
	n = 1 (25%-OIT)	n = 1 (25%-OIT)	n = 1 (25%-OIT)
Children disliking the food	n = 5 (25%-OIT)		
Sudden termination of hospital visits	n = 3 (100%-OIT)	n = 1 (100%-OIT)	n = 2 (100%-OIT)
	n = 1 (25%-OIT)	n = 1 (25%-OIT)	
Enrolling in a different OIT study	n = 1 (25%-OIT)		
Worsening of atopic dermatitis			n = 1 (25%-OIT)
Parental anxiety		n = 1 (25%-OIT)	
Loss to follow-up by other reasons			
Not extended follow up to the 2nd year	n = 6 (100%-OIT)	n = 4 (100%-OIT)	n = 1 (100%-OIT)
	n = 1 (25%-OIT)		
Moving away		n = 1 (100%-OIT)	
Acute infection		n = 1 (25%-OIT)	
Mother's pregnancy	n = 1 (25%-OIT)		

Supplemental Figures

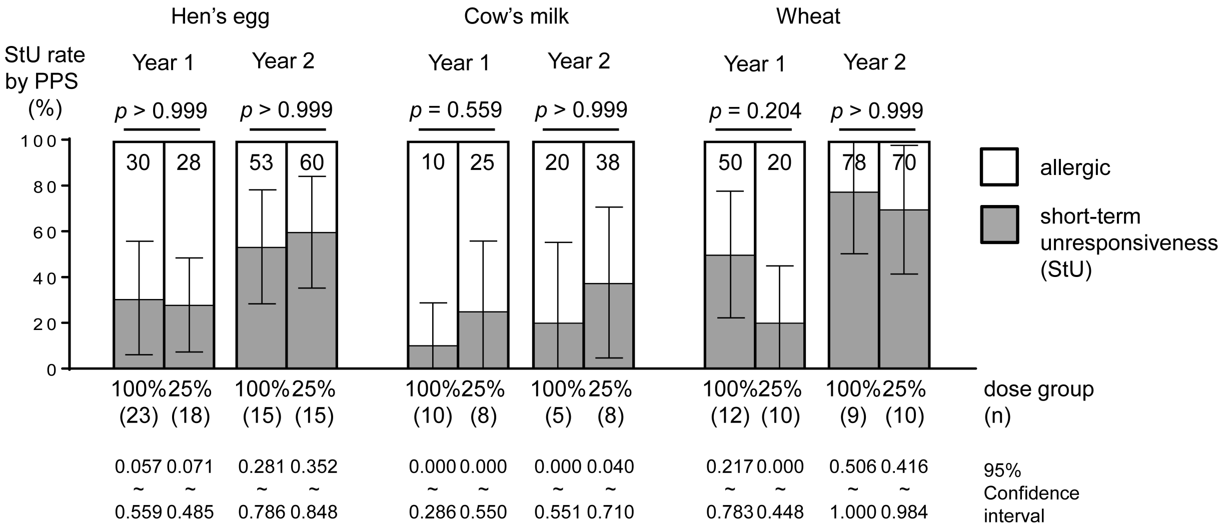

Fig. S1. Comparison of outcome between 100%-dose group and 25%-dose group.

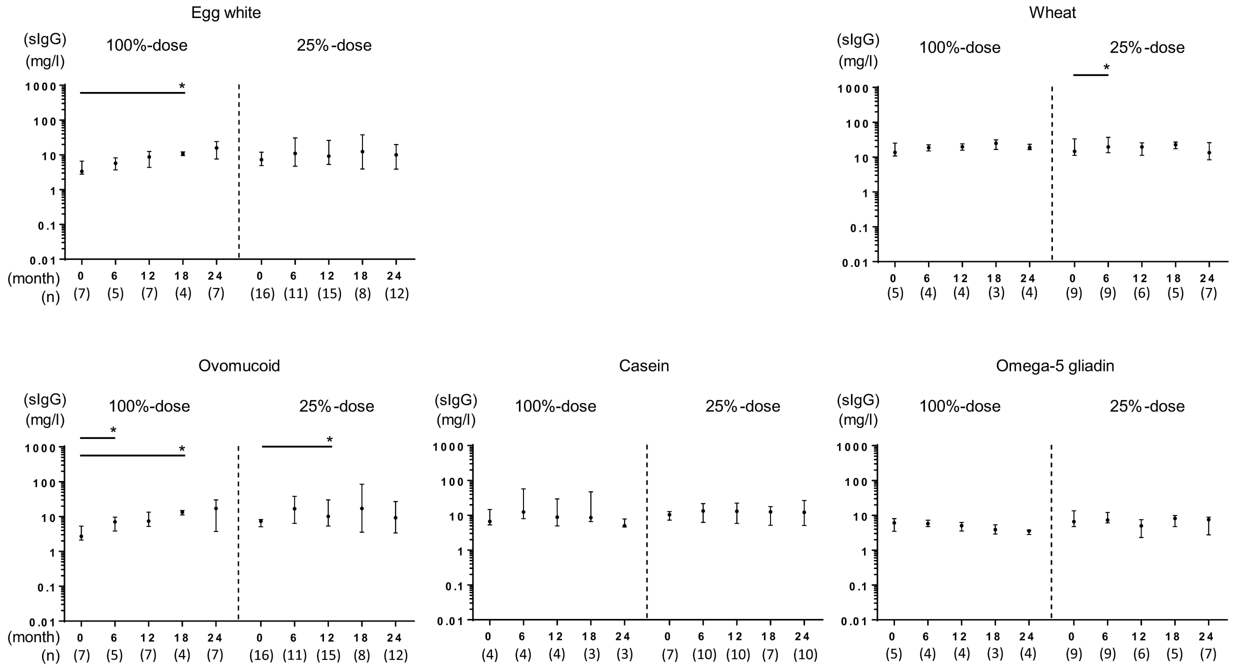

Fig. S2. Immunological changes of sIgG during OIT.

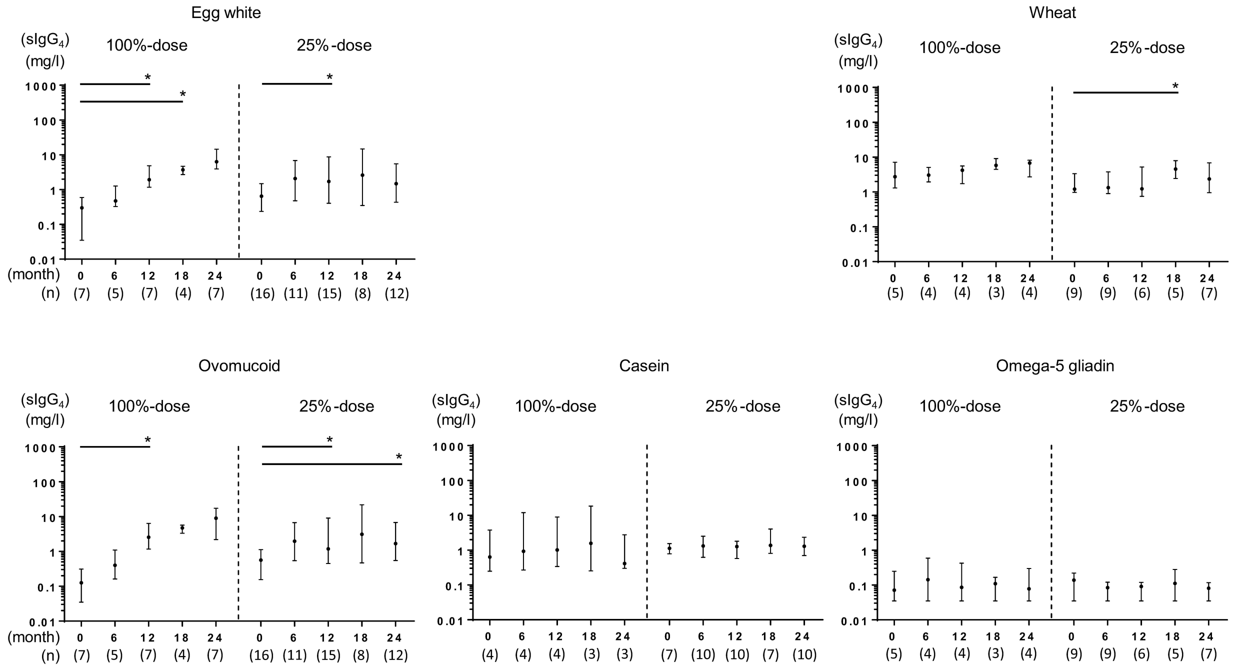

Fig. S3. Immunological changes of $sIgG_4$ during OIT.

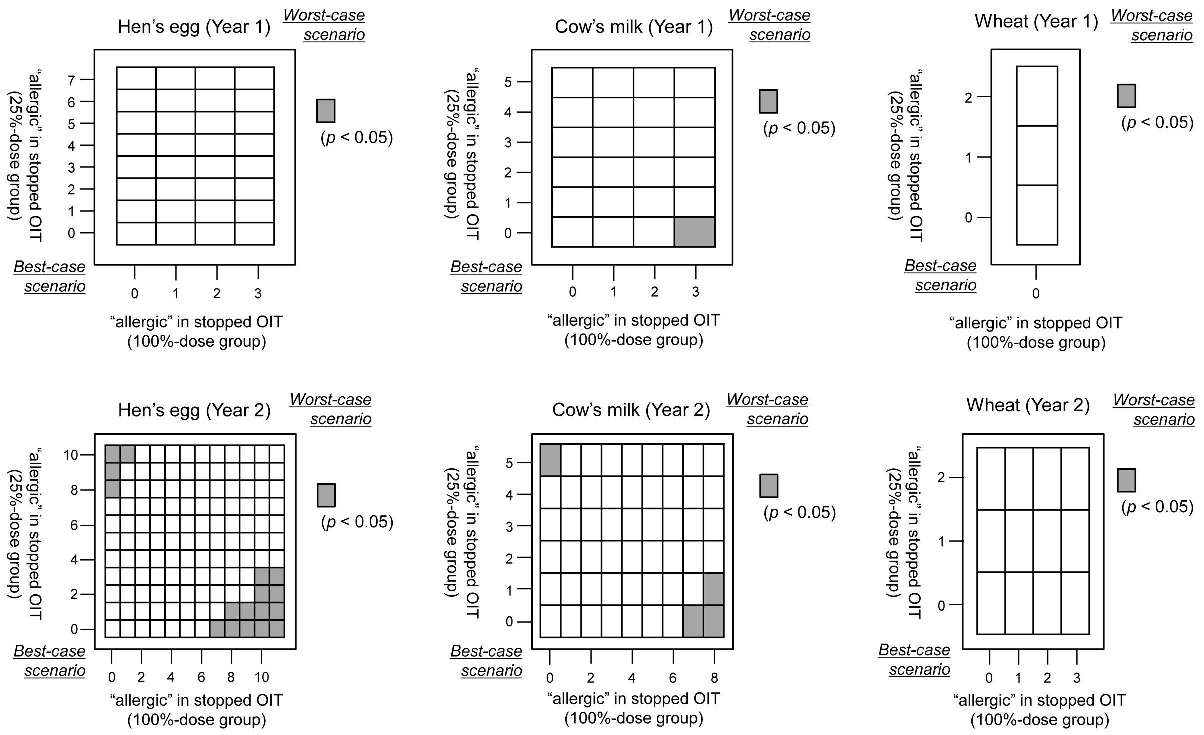

Fig. S4. Tipping-point analysis of outcome between the 100% and 25% dose groups in years 1 and 2.
